# ACTH Treatment for Management of Nephrotic Syndrome: A Systematic Review and Reappraisal

**DOI:** 10.1155/2020/2597079

**Published:** 2020-06-04

**Authors:** Ronith Chakraborty, Arul Mehta, Nikhil Nair, Lena Nemer, Rahul Jain, Hirva Joshi, Rupesh Raina

**Affiliations:** ^1^Akron Nephrology Associates/Cleveland Clinic Akron General, Akron, OH, USA; ^2^Summer Research Student, Akron Nephrology Associates/Cleveland Clinic Akron General, Akron, OH, USA; ^3^Department of Chemistry, Case Western Reserve University, Cleveland, OH, USA; ^4^Harvey S. Firestone High School, Akron, OH, USA; ^5^Northeast Ohio Medical University, Rootstown, OH, USA; ^6^Department of Nephrology, Akron Children's Hospital, Akron, OH, USA

## Abstract

**Background:**

In recent years, the use of adrenocorticotropic hormone (ACTH) therapy for treatment of proteinuria due to nephrotic syndrome (NS) has been heavily explored. ACTH therapy, which comes in the natural (H. P. Acthar Gel) or synthetic (tetracosactide) form, has resulted in remission in patients with immunosuppressive and steroid-resistant NS. However, the exact efficacy of ACTH therapy in the NS etiologies, such as membranous nephropathy (MN), focal segmental glomerulosclerosis (FSGS), minimal change disease (MCD), lupus nephritis (LN), IgA nephropathy (IgAN), and membranoproliferative glomerulonephritis (MPGN), has not been determined.

**Objective:**

This systematic review analyzed the published literature on ACTH therapy in various NS etiologies to determine its efficacy.

**Methods:**

A comprehensive search of MEDLINE, EMBASE, and Cochrane databases was conducted for articles through June 2019. An additional search was performed on clinicaltrials.gov to search for additional trials and cross reference the results of our database search. The literature which studied synthetic or natural ACTH treatment in patients with known etiologies of NS was included. Studies were excluded when they consisted of a single case report or did not analyze the lone effect of ACTH in NS.

**Results:**

The initial search yielded a total of 411 papers, and 22 papers were included. In 214 MN patients, there was an overall remission of 40% (85/214) and an overall remission of 43% (42/98) in FSGS patients. In other etiologies, there were overall remissions of 78% (11/14), 31% (5/16), 40% (16/40), and 62% (8/13) in MCD, LN, IgAN, and MPGN patients, respectively.

**Conclusion:**

ACTH showed benefits in proteinuria reduction across all etiologies of NS. However, more randomized controlled studies with larger population sets and longer follow-ups are imperative to establish causal benefits. New studies into its efficacy in children are also necessary.

## 1. Introduction

Adrenocorticotropic hormone (ACTH), a pituitary polypeptide hormone consisting of 39 amino acids, plays a pivotal role in the hypothalamic-pituitary-adrenal (HPA) axis and is crucial in maintaining homeostasis in the neuroimmune-endocrine system [1]. H. P. Acthar® Gel, a form of ACTH therapy, is a highly purified form of ACTH and is delivered as a gel to provide extended release of ACTH following injection. It was initially FDA-approved in 1952 for the reduction of proteinuria and hyperlipidemia associated with childhood nephrotic syndrome (NS). However, given its high cost and subcutaneous administration, it was replaced by synthetic oral glucocorticoids, a cheaper and more convenient treatment [1]. Thus, steroids have become the first line of treatment [2–7]. However, 10–20% of patients fail to respond to initial steroid treatment due to steroid resistance caused by genetic mutations [1]. Therefore, the search to find treatment options that operated via nonsteroidal pathways began. In 1999, Berg et al. began a reexamination of ACTH therapy, which lead to a multitude of trials investigating the extent of its benefits [8]. Since then, the literature has expanded on the use of ACTH therapy in various etiologies of NS. The purpose of this systematic review is to examine current literature in order to critically appraise the efficacy, safety profile, and extent of benefits that ACTH therapy provides to patients with multiple etiologies of NS. Given the paucity of large-scale studies, we present the compilation of smaller scale studies in order to spur the creation larger studies.

## 2. Mechanism of Action

**Figure 1:**
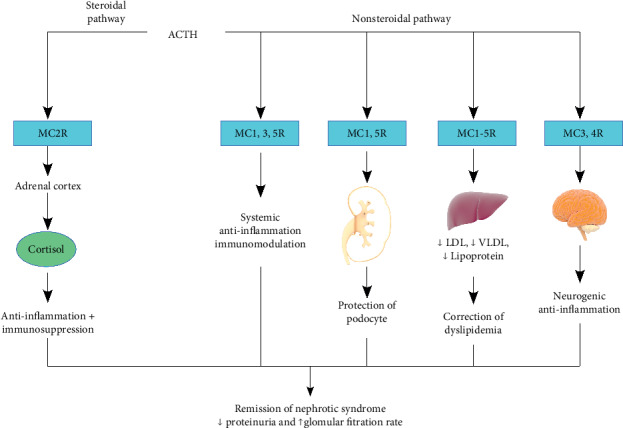
ACTH as a melanocortin receptor antagonist. Adapted from Gong [9].

**Figure 2:**
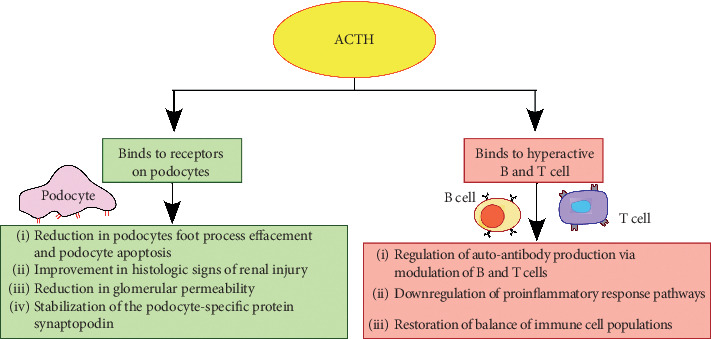
ACTH binding to receptors on the kidney and immune cells [9, 11].

ACTH is believed to play a role as an antagonist of the melanocortin system by binding to all 5 melanocortin receptors (MCRs) (Figure 1) [9]. This information has been postulated based on nonclinical data from several studies and is being further investigated. MCRs have been found to be expressed in a variety of cells, including podocytes, glomerular cells, and multiple immune cells, and play an active role in anti-inflammation, lipolysis, and modulation of exocrine function [9, 10]. ACTH has shown to directly bind to receptors on podocytes and lead to stabilization of the podocyte specific protein synaptopodin, reduction in podocyte foot process effacement and apoptosis, improvement in histological signs of renal injury, and reduction in glomerular permeability (Figure 2) [9, 11]. Additionally, several animal studies have suggested that the ACTH-MCR interaction in glomerular podocytes and renal parenchymal cells leads to a subsequent decrease in proteinuria. In a study by Lindskog et al., rats raised with passive Heyman nephritis and given synthetic ACTH exhibited large reductions in proteinuria that were correlated with improved renal physiology confirmed via biopsy [10]. Furthermore, ACTH has shown modulatory effects of the immune response. In specific, binding of ACTH to hyperactive immune cells has shown to modulate hyperactive T and B lymphocytes resulting in the regulation of autoantibody production, restore the balance of immune cell populations, and downregulate the proinflammatory response pathways to minimize the expression of inflammatory mediators [9, 11]. ACTH is also involved in clearing anti-PLA2R antibodies. PLA2R antibodies are expressed in glomerular podocytes, causing both inflammation and death of surrounding renal tissue and leading to increased proteinuria [9]. An alternate mechanism of note is through apolipoprotein metabolism. ACTH may recover the glomerular expression of clusterin (apolipoprotein J) as the C5b-9 component competes with clusterin for the megalin receptor in podocytes. The heightened levels of clusterin could act as a competitive inhibitor and decrease binding by complement [12]. In contrast, glucocorticoids do not bind to MCRs; instead, they bind to receptors in the cytoplasm, which can upregulate the expression of anti-inflammatory proteins [1]. Lastly, in a randomized, pharmacodynamic study by Lal et al., it was found that the cortisol-equivalent exposure for 80 units twice weekly of ACTH equated to 75 mg of prednisone weekly [13]. The lower cortisol equivalency of ACTH found in comparison to intravenous methylprednisolone suggests that ACTH work through various other mechanism, rather than solely effecting the adrenal glands [13].

## 3. Methods

### 3.1. Literature Search

Two reviewers searched PubMed/Medline (1946 to 2019), Embase (1974 to 2019), and Cochrane Database to include all publications involving ACTH and NS. Medical subject headings (MeSH) were used in the search strategy and included nephrotic syndrome, adrenocorticotropic hormone, adrenocorticotropi*∗* adj3 hormone, *∗* acth, corticotropin, and *∗* adrenocorticotropin*∗*. The asterisk (*∗*) allowed for truncation searching and broadened the search to include various word endings. All literature was reviewed individually by two reviewers. A third independent reviewer resolved any disagreements in data extraction. The development of the search strategy involved the assistance of a research librarian and was restricted to the English language. The full search strategy is listed in Table 1. These results were cross examined with a search on http://www.clinicaltrials.gov to search for additional trials and those that were excluded are detailed in Table 2.

### 3.2. Selection Criteria

Studies were eligible for inclusion when referencing ACTH treatment in either its synthetic or natural form, reporting known etiologies of NS. Data also needed to mention the efficacy or safety of ACTH treatment in the etiologies. Additionally, randomized controlled trials and observational studies were part of the inclusion criteria. Single case reports and studies that did not analyze the effects of ACTH in NS alone were excluded. The selection criteria is described in the PICOS model (Table 3).

### 3.3. Data Extraction

A standardized data collection form was used to extract the following information from each included study: last name of first author, study protocol, publication year, and patient population studied. Characteristics of participants included type of glomerular disease, form of ACTH preparation, dosage, duration of treatment, treatment response and type, and complications following ACTH treatment.

## 4. Results

### 4.1. Results of Literature Search

**Figure 3:**
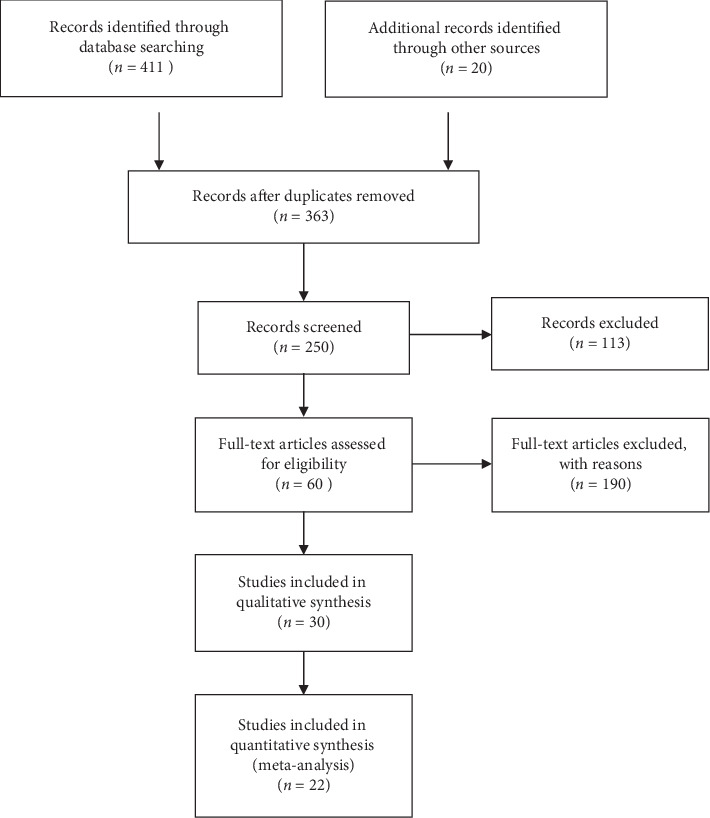
PRISMA flowchart showing studies considered for inclusion.

A flow diagram for retrieval and inclusion followed PRISMA guidelines (Figure 2). Our search strategy yielded 411 potentially relevant articles. The search on clinicaltrials.gov provided an additional 20 articles. A total of 371 articles were excluded after the initial screening, and 60 articles were included for full-length review (Figure 3). Eventually, 21 articles met the inclusion criteria. Details of these included studies are outlined in Tables 4–10.

### 4.2. Efficacy of ACTH in the Treatment of Glomerular Diseases

A total of 21 studies were included, with an even mix of retrospective and prospective studies, to evaluate ACTH as a treatment option (*n* = 342). The two most prevalent NS etiologies were membranous nephropathy (MN) and focal segmental glomerulosclerosis (FSGS). Less common but significant etiologies included minimal change disease (MCD), IgA nephropathy (IgAN), lupus nephritis (LN), and membranoproliferative glomerulonephritis (MPGN).

Patient manifestations from the studies varied in terms of characteristics, disease severity, ACTH preparations, dosing, treatment, and response criteria. In order to standardize the differing protocols, all results were indicated based on trial definition, and the absolute values of all provided lab results were displayed to show mean change after treatment. For the majority of patients, complete remission was defined as having proteinuria of ≤0.3 g/day. Partial remission was defined mostly as ≥50% reduction in proteinuria along with <3.5 g/day. Patients who did not cross these thresholds were categorized primarily as nonresponders even when proteinuria was seen. As many of these trials were retrospective in nature, control groups and baseline lab values are missing or not reported. Given their scale at which these studies have been conducted, there could be potential for publication bias.

### 4.3. Membranous Nephropathy

A total of 12 studies analyzed 172 patients diagnosed with MN (Table 5) [2, 4, 5, 7, 8, 14–20]. The dosage of synthetic ACTH varied, ranging from 0.25 mg/week to 2.25 mg/week. Natural ACTH of 80–160 units was administered with treatment lengths varying from 2–24 months. The maximum duration was 82 months based on studies that reported follow-ups. The complete remission rate was 20% while the partial remission rate was 33%.

In a study by Berg et al., 14 patients were treated with increasing doses of synthetic ACTH, Tetracosactide (Synacthen Depot), for 56 days. The optimal dosage was 1 mg twice per week where a 90% reduction in serum albumin excretion, and a 25% increase in glomerular filtration rate was seen [8]. Out of the 14 patients, one achieved complete remission and 13 achieved partial remission [8]. In a second randomized trial by Poticelli et al., one year of synthetic ACTH analog (*n* = 16) was compared with alternative months of the corticosteroid methylprednisolone with an alkylating agent, cyclophosphamide (*n* = 16), for six months [14]. There was no significant difference in remission rates (14 vs 15 patients) [14]. During the follow-up period, however, patients treated with ACTH achieved eight complete and six partial remissions in contrast to the four and eight remissions, respectively, in the methylprednisolone/cyclophosphamide group [14]. Another study by Lorusso et al. involved administering synthetic ACTH (1 mg/wk) to nine patients who had previously received immunosuppressive treatments (high-dose prednisone, cyclophosphamide, cyclosporine, and mycophenolate mofetil) for 12 months [15]. There was a reduction of proteinuria from 5.3 g/day to 1.52 g/day with four patients achieving complete remission [15]. Van De Logt et al. studied 20 patients being treated with synthetic ACTH (tetracosactide) and compared them with cyclophosphamide and steroid treatment (control) [16]. Patients treated with ACTH resulted in four complete and seven partial remissions compared with patients treated with cyclophosphamide (13 complete and 6 partial remissions) [16]. This indicates that synthetic ACTH is less effective in inducing remission than cyclophosphamide [16]. Patients treated with synthetic ACTH reported more adverse effects including mood disorders, increasing edema, myalgia, fever/infection, skin hyperpigmentation, hypokalemia, and cushingoid face [16].

Three studies examined the effects of different dosages of H. P. Acthar gel (natural) in MN patients. Hladunewich et al. treated patients with 40/80 units of Acthar with total amounts equaling 800, 1,760, or 2,800 units [19]. Patients who received increasing amounts of Acthar had increased remission of proteinuria [19]. Patients administered 80 units weekly of Acthar did not have significant improvement in proteinuria, thus indicating a minimum amount needed to observe clinically relevant responses. Additionally, there was a statistically significant correlation between lower anti-PLA2R antibodies and decreased proteinuria with antibody clearance occurring before (*n* = 5) or parallel (*n* = 2) in 15 patients with detectable antibody levels over 12 months. Most ACTH studies used 2 mg/week dosages, but lower amounts at 1 mg/week resulted in response rates of at least 40% [19]. In a different study by Madan et al., 11 patients were treated with 80 units of Acthar twice weekly for six months and resulted in a 61.1% reduction in proteinuria in 10 patients, with two achieving complete and four achieving partial remission [2]. Additionally, Bomback et al. reported a mean reduction in proteinuria levels from 6.83 g/day to 2.85 g/day study with administration of 160 units/week of Acthar for 6 to 12 months. Minor adverse effects (AEs) included hyperglycemia (*n* = 2), weight gain (*n* = 1), and bone demineralization (*n* = 1) [4]. Bomback et al. varied the dosing of Acthar in another study, with five patients receiving 40 units twice a week for two weeks following 80 units twice a week for 24 weeks. This resulted in a smaller reduction in mean proteinuria from 5.65 mg/g to 4.42 mg/g with only two partial remissions [5].

The recent Acthar for Treatment of Proteinuria in Membranous Nephropathy patients (CHARTs) study examined the efficacy of 40 units vs. 80 units of Acthar to a placebo group [20]. The primary outcome was complete or partial remission after 8 treatment cycles (classified as = UPCR (urea protein creatinine ration) < 0.3 g/g and = UPCR < 50% of baseline UPCR and >0.3 g/g but <3.0 g/g, respectively). One patient observed partial remission in the 40-unit arm, whereas five patients observed partial remission in the 80-unit arm with nonsignificant difference in AEs amongst all trial groups [20].

### 4.4. Focal Segmental Glomerulosclerosis (FSGS)

Nine studies analyzing 98 patients with FSGS were included (Table 6) [2–5, 15, 21–24]. The dosage of synthetic ACTH administered ranged from 0.5 to 1 mg/week while natural ACTH was prescribed in doses of 40 or 80 units with treatment lengths varying from 6 to 12 months. In the study by Madan et al., a 57.7% reduction in proteinuria from baseline (mean reduction 3,021.7 ± 1,970 mg/d, *P* < 0.0001) was observed [2]. No patients achieved complete remission; however, nine had partial remission and four noted some proteinuria reduction (range 31.6–42.4%). Early termination occurred in two patients, one due to swelling [2]. Tumlin et al. studied 13 patients treated with ACTH who had failed to respond to steroid therapy and calcineurin inhibitors [21]. A reduction in UPCR ratio from 7.92 ± 1.1 g/g to 2.98 ± 0.6 g/g (*P*=0.0005) was observed. One patient attained complete remission, while eight achieved partial remission [21].

The following studies used natural ACTH. In 2013, Hogan et al. studied 24 steroid-dependent or resistant patients undergoing various Acthar treatments: Stanford regimen (2,160 units), Columbia regimen (3,840 units), or individualized treatment regimen (80 units twice weekly) [22]. Two patients achieved complete remission (*n* = 1 Stanford, *n* = 1 Columbia) and five patients achieved partial remission (*n* = 2 Stanford, *n* = 1 Columbia, *n* = 2 Individual) [22]. There was a significant reduction in median proteinuria levels from 4,595 mg/g to 2,243 mg/g. However, 21 patients experienced swelling, mood alterations, upper respiratory infection, dyspepsia, hyperglycemia, muscle cramps, polyurea, and elevated blood pressure [22]. Alhamad et al. studied 20 patients treated with 80 units of Acthar gel, resulting in four complete and six partial remissions [23]. In a different study by Bomback et al., one of three patients achieved partial remission with treatment of 40 units BIW for two weeks following 80 units BIW for 24 weeks [4]. Filippone et al. examined 10 patients treated with 40 units weekly to 80 units twice weekly of Acthar gel [3]. Two patients had partial remission, while another two achieved complete remission. Patients experienced weight gain (*n* = 2), myalgia (*n* = 2), worsening diabetes (*n* = 2), hypertension (*n* = 2), and edema (*n* = 1) but no individuals stopped treatment [3].

Two studies analyzed synthetic ACTH. In one study, Berg et al. found that treatment with synthetic ACTH decreased mean urinary albumin from 3,400 mg/d to 1,700 mg/d and resulted in one partial remission [25]. Lorusso et al. likewise showed that patients receiving Tetracosactide had a decrease in proteinuria from 18 g/d to 0.93 g/d. No adverse effects were reported for the two synthetic ACTH studies [15]. In summary, 33 patients (33.6%) observed partial remission, nine patients (9.2%) observed complete remission, and six patients (6.1%) observed some reduction in proteinuria.

### 4.5. Minimal Change Disease (MCD)

Seven studies included 14 patients diagnosed with MCD (Table 7) [2–7, 15]. Ten of those patients were treated with Acthar while four were treated with synthetic ACTH. After a mean follow-up of six months, the overall, partial, and complete response rates were 78.5%. Acthar doses varied between 40–80 units for 24 weeks. A 2016 study by Madan et al. (*n* = 2) noted a 98.1% reduction in proteinuria and a mean serum increase of 0.60 ± 0.6 g/dl in patients given 80 units of Acthar twice weekly for six months. Both patients experienced complete remission [2]. Filippone et al. studied three patients who were previously treated with prednisone with one patient additionally treated with cyclophosphamide. One patient achieved complete remission, while two achieved partial remissions with a significant reduction in proteinuria [3]. Bomback et al. found that 80 units twice weekly of Acthar for four months resulted in no effect on a patient's proteinuria [4]. However, a subsequent study by Bomback et al. found that 1 of 2 patients achieved partial remission when treated with a varied dose (40 units twice weekly for 2 weeks, then 80 units afterwards for 24 weeks) [5]. Lastly, a study by Khastgar et al. reported that two patients receiving 80 units of Acthar both achieved complete remission within six months [6].

Doses for synthetic ACTH varied between 1 and 2 mg/wk for 12 months. A study by Berg and Arnadottir showed that two patients achieved complete remission and a reduction in mean proteinuria from 6,747 mg/day to 277 mg/day [7]. Similarly, Lorusso et al. 2015 (*n* = 2) showed that synthetic ACTH analog, tetracosactide, resulted in one complete remission with proteinuria reduction from 3.0 g/day to 0.5 g/day [15].

### 4.6. Lupus Nephritis (LN)

Of the four studies included, 15 patients were treated with varying doses of 40/80 units of Acthar gel twice weekly for 1–6 months (Table 8) [2, 5, 6, 16, 26]. Khastgir et al. treated two patients with 80 units of Acthar gel twice weekly resulting in partial remission for both [6]. Similarly, a study by Madan et al. (*n* = 2) resulted in an 87.3% reduction rate in proteinuria with both patients achieving partial remission [2]. A 2014 study (*n* = 10) by Feichtner and Montroy administered 80 units of Acthar for a shorter period of time (7–15 days) and found a significant improvement in joint and active skin problems with no serious or unexpected adverse effects. However, one patient experienced bilateral edema in the legs for two weeks [26]. In total, four patients (27%) observed partial reduction in proteinuria while all patients observed reductions in flares and secondary symptoms of lupus nephropathy.

### 4.7. IgA Nephropathy (IgAN)

Throughout five studies, 35 patients were diagnosed with IgAN (Table 9) [2, 4–6, 24]. All patients were treated with 40–160 units of Acthar for a minimum of 6 months. In a study by Bomback et al. (*n* = 1), the patient was treated with 40 units twice per week, resulting in complete remission with a decrease in proteinuria from 4,952 mg/day to 42 mg/day [5]. Bomback et al. later conducted another study which resulted in one patient achieving complete remission, one achieving partial remission, and three nonresponders with some reduction in proteinuria [4]. One patient withdrew due to worsening kidney function. In a similar sized study by Madan et al. (*n* = 5), patients were treated with 160 units/week of natural Acthar for at least six months, which resulted in two partial remissions with a 67.3% reduction in mean proteinuria (fell from 4,268 ± 2,931 mg/d to 1,416 ± 654 mg/d) as a result of the administration of 80 units twice weekly of Acthar gel [2]. Similarly, a study by Khastgar et al. resulted in two patients achieving partial remission; however, a 36.4% reduction in proteinuria was found at the same dosage with one patient engaging in early termination due to hypertension and weight gain [6].

### 4.8. Membranoproliferative Glomerulonephritis (MPGN)

Throughout four studies, 13 patients were diagnosed with MPGN, seven achieved complete remission, and one achieved partial remission (Table 10) [2, 5, 7, 15]. Five patients were treated with 40–80 units of Acthar, while eight patients were treated with 1-2 mg of synthetic ACTH. In the study by Berg and Arnadottir (*n* = 6), patients were treated with synthetic ACTH resulting in a decrease in mean proteinuria from 12.04 ± 7.8 g/day to 0.392 ± 0.304 g/d and complete remission [7]. Lorusso et al. (*n* = 2) found a decrease in mean proteinuria from 13 g/day to 5.8 g/day [15]. One patient experienced complete remission, while the other underwent early termination. Bomback et al. (*n* = 4) treated three patients with 80 units twice per week, while one patient was treated with 40 units three times per week [5]. The 80-unit group had no response; however, the patient treated with 40 units showed a limited response with a decrease in proteinuria from 12.40 g/d to 4.56 g/day [5]. In another study by Madan et al., the patient received 80 units of Acthar gel twice weekly and achieved partial remission with a 78.6% reduction in proteinuria (from 10,000 mg/d to 2141 mg/d) [2].

## 5. Discussion

This systematic review evaluates the effectiveness of ACTH in the treatment of various NS etiologies. There are two forms of ACTH injections currently available. One is natural ACTH, sold as H. P. Acthar gel (Mallinckrodt). The other is a synthetic form of ACTH, called Tetracosactide (Synacthen Depot), which contains only the first 24 amino acids of the ACTH polypeptide but retains full functionality. Both forms of ACTH treatment have shown to perform the same biological function of naturally occurring ACTH, both acting through steroid-independent pathways, as evident by their effectiveness in eliciting remission of proteinuria in steroid-resistant patients. The selected studies in our review showed a promising correlation between the use of ACTH and its reduction of proteinuria and significant benefits in MN and FSGS in comparison to other etiologies.

For the two most common etiologies, MN and FSGS, current treatment includes the use of immunosuppressive therapies, such as alkylating agents, calcineurin inhibitors (CNIs), mycophenolate mofetil (MMF), and rituximab [27–32]. On average, we found reduced proteinuria across both etiologies with remission rates of 70% and 42%, respectively, after ACTH treatment. In a study by Lorusso et al., the authors evaluated the effect of ACTH on nine MN patients previously treated with immunosuppressive and showed significant reduction in proteinuria with four patients achieving complete remission [15]. In regard to FSGS patients, Tumlin et al. illustrated that 70% of patients who were steroid resistant achieved complete or partial remission [21]. Additionally, Hogan et al. evaluated 24 patients who were steroid resistant or steroid dependent and previously failed at least two immunosuppressive treatments [22]. The patients underwent various dosing of ACTH therapy and had a cumulative remission rate of 29%. The remission rate was lower in comparison to other studies, and the authors suggest that the low remission rate may be due to heterogeneity in FSGS pathogenesis and genetic causes of FSGS that may be unresponsive to treatment [22]. However, despite the paucity in subjects and the retrospective nature of the studies, it was clear that the largest benefits were seen in patients who were steroid resistant. It also appears that higher dosage of ACTH for longer duration of time, such as 2 mg/week synthetic or 160 units of natural ACTH for nine months, leads to higher rates of remission and lower rates of future relapse [18, 19]. However, larger patient studies are needed to better confirm this theory and there is a strong correlation to suggest that high dosage initially is the most beneficial option.

Additionally, MCD has showed similar responses as MN and FSGS with a statistically significant (10–20%) population becoming resistant to standard steroid treatments. Alternate treatments include cyclophosphamide, cyclosporine, tacrolimus, and MMF [33–35]. Studies with 2-3 mg/kg/day of cyclophosphamide administered for 8–16 days have shown overall remission rates of 63%–86% [34]. In a study by Madan et al., the authors showed a reduction of 98.1% in proteinuria with both patients showing complete remission [2]. Additionally, Khastgir et al. (*n* = 2) had two complete remissions using 80 units of Acthar, while Berg and Arnadottir had 100% complete remission (*n* = 2) using synthetic ACTH [6]. Overall, ACTH therapy in MCD showed to be advantageous and showed promise with the majority (*n* = 7) of patients showing partial or complete remission. While the rates of response are encouraging, larger scale studies are necessary to assess remission rates in a sizeable patient cohort.

Furthermore, alternative treatments are also needed for patients with LN, which result from moderate to severe systemic lupus erythematosus (SLE) and resistance or ineffectiveness to traditional treatments. Standard of care for SLE currently includes corticosteroids, such as prednisone, and immunosuppressive drugs, such as cyclophosphamide, and MMF [36, 37]. However, the use of these treatments can have severe adverse effects, including an increased risk of infections, diabetes, increased blood pressure, and fluid retention. Cyclophosphamide and abatacept have been tested together in the Abatacept and Cyclophosphamide Combination Efficacy and Safety Study (ACCESS) trial, which looked at the side effects of blocking the CD28/CD80 costimulatory pathway in LN with abatacept, a CTLA4-Ig construct [37]. Abatacept did not offer any further benefit for induction of remission when added to low-dose cyclophosphamide [37]. ACTH is also an FDA-approved therapeutic for SLE, but the paucity of clinical data regarding its effectiveness is limited. The administration of ACTH following corticosteroid and immunosuppressive therapy was shown to be effective in a study by Fiechtner and Montroy. All patients receiving standard therapy with Acthar gel showed significant reduction in intensity of flares with no serious side effects [26]. Although these patients had moderately to severely active SLE and were undergoing treatment with traditional therapeutic agents, the subsequent treatment with Acthar led to significant improvements. Additionally, partial reduction in proteinuria was noted in five patients (31%) after ACTH treatment in a retrospective study by Li et al. [38]. The results suggest that ACTH therapy may provide a novel anti-inflammatory and immunomodulatory treatment option. Although more data are needed, ACTH seems to be a promising agent for reduction of proteinuria and SLE flares.

ACTH therapy has also been considered as an alternate treatment for IgA nephropathy. In our review, there was an overall remission rate of 46% in 35 patients treated with ACTH. Bomback et al. reported two patients with resistant IgA nephropathy and showed greater than 50% reduction in proteinuria (one complete and partial remission) [4]. In a study by Madan et al., it was showed that two out of five patients achieved partial remission and there was a 67.3% reduction in proteinuria [20]. However, there were four early termination due to adverse effects such as weight gain, hypertension, and worsened kidney function. Even though these studies demonstrated a reduction in proteinuria, more large-scale investigations are needed to evaluate the efficacy of ACTH treatment in IgA nephropathy patients.

Furthermore, ACTH in our review had fewer and less extreme adverse side effects in comparison to the current standard of care for NS. Edema was the most common sequelae seen and was often times easily treated with diuretics. In addition, insomnia and mood swings were other commonly seen side effects. The pathophysiology behind these sequelae is unclear, although it may be due to neurobiological effects from the melanocortin system activation [39]. Additional studies which directly compare the safety of ACTH with that of oral glucocorticoids are necessary to better understand how specific harms arise and whether certain etiologies or treatments exacerbate their occurrence. There are very few RCTs on corticosteroid or other immunosuppressive use in patients with NS. Small, observational studies with short-term follow-up have shown some benefits of immunosuppressive therapy (cyclophosphamide or MMF) combined with high-dose IV or oral steroids mostly in subjects with a rapidly progressive condition specifically with extreme proteinuria [39]. Given the effectiveness of ACTH with steroid resistant patients, these studies seem to provide credence to ACTH being able to operate through nonsteroidal pathways. While no consensus can be delineated given the nature of these studies, they do show promise of efficacy.

### 5.1. Limitations

This review harbors some limitations. Most studies were small in size with none having more than 60 patients for any given etiology. MN and FSGS were by far the most common etiologies seen but still suffer from small sample sizes. Etiologies such as MCD, LN, MPGN, and DN had even smaller study populations, most with 1–5 patients per study. Given that the majority of these trials were done retrospectively, it is hard to derive definitive conclusions. The small size of these studies and with mostly favorable results shown, there is a possibility of publication bias with a sizeable number of trials being sponsored by Mallinckrodt. Very few of the studies analyzed were prospective in nature and as such, it could not be determined whether or not there was allocation bias on part of the clinicians who initially provided the treatment. Thus, more randomized controlled studies of larger populations are necessary to compare both treatment duration and the effect of different treatment options in order to truly delineate the benefits of ACTH therapy over the current standard of care.

## 6. Conclusion

In conclusion, ACTH shows benefits in proteinuria reduction across all etiologies of NS with its most substantial impact seen in MN. It exhibits mild adverse side effects and has strong follow-up numbers in patients who have previously suffered from immunosuppressive and steroid resistance. More randomized controlled studies with larger population sets and longer follow-up are imperative to establish direct benefits of ACTH therapy. Furthermore, given ACTH's history as a treatment in pediatric NS, new studies into its efficacy in children should be investigated [25].

## Figures and Tables

**Table 1:** Search strategy.

Database	Medline
Date	06/04/2019
Strategy	
	(1) Nephrotic syndrome/ (2) “nephrotic syndrome^*∗*^.”tw. (3) 1 or 2 (4) exp adrenocorticotropic hormone/ (5) ((adrenocorticotropi^*∗*^ adj3 hormone^*∗*^) or acth or corticotropin^*∗*^ or adrenocorticotropin^*∗*^) mp. (6) 4 or 5 (7) 3 and 6 (8) Limit 7 to English language

Database	Embase

Date	06/04/2019
Strategy	
	(1) exp nephrotic syndrome/ (2) “nephrotic syndrome^*∗*^.”tw. (3) 1 or 2 (4) corticotropin/ (5) ((adrenocorticotropi^*∗*^ adj3 hormone^*∗*^) or acth or corticotropin^*∗*^ or adrenocorticotropin^*∗*^) mp. (6) 4 or 5 (7) 3 and 6 (8) Limit 7 to English language

Database	Embase

Date	06/04/2019
Strategy	
	(1) mh “nephrotic syndrome”] (2) “nephrotic syndrome^*∗*^” (3) #1 or #2 (4) [mh “adrenocorticotropic hormone”] (5) (adrenocorticotropi^*∗*^ near/3 hormone^*∗*^) or acth or corticotropin^*∗*^ or adrenocorticotropin^*∗*^ (6) #4 or #5 (7) #3 and #6 in trials

**Table 2:** Summary of clinical trials found on http://www.clinicaltrials.gov.

Study	*N*	Protocol	Status	Outcome
ACTHAR gel for drug-resistant nephrotic syndrome in children (ADRENL)	0	Acthar gel will be dosed by body surface area (BSA) using the dubois method. The dose of Acthar gel will be 80 units/1.73 m^2^ per dose administered subcutaneously (SQ) twice a week.	Completed	N/A
Pilot study of acthar® gel in chronic inflammatory demyelinating neuropathy	0	For the first two weeks, participants will receive 1 mL of study drug subcutaneously every other day. After that, participants will receive 1 mL of study drug twice a week for up to 6 months.	Completed	N/A
Acthar as rescue therapy for transplant glomerulopathy in kidney transplant recipients	2	Acthar 80 units twice weekly for 6 months. If endpoint is not reached, duration may be increased to 12 months.	Completed	N/A
ACTHAR GEL in patients with membranous (class V) lupus nephritis	0	Randomized parallel assignment, 80 IU administered subcutaneously BIW for 6 months.	Completed	N/A
ACTH treatment of APOL1-associated nephropathy	0	Acthar 40 units subcutaneously three times a week or 80 units twice a week. Nonrandomized.	Completed	N/A
Acthar on proteinuria in IgA nephropathy patients	0	Randomized allocation of 80-unit injections 2x weekly.	Completed	N/A
Use of acthar in patients with FSGS that will be undergoing renal transplantation	3	Patients received acthar to measure rate of recurrence of FSGS after transplant.	Completed	N/A
Safety and efficacy of acthar gel in an outpatient dialysis population	9	Randomized with parallel assignment and double masking. Subjects given either 80 or 40 units subcutaneously 2x weekly.	Completed	N/A
Acthar SLE NYU langone health	0	Acthar gel: 40–80 units, once per day	Completed	N/A
Comparative and efficacy study of acthar gel alone or in combination with tacrolimus in fibrillary glomerulopathy (fact)	34	Nonrandomized, single group treatment. ACTHar gel alone-patients will be receive 80 units SQ 2X/week for 52 weeks. ACTH gel 80 units 2X per week plus oral tacrolimus (1.0 mg·BID) titrating to a trough level of 4–6 ng/ml for 52 weeks.	Ongoing	Change in UP/Cr ratio in patients with biopsy-proven fibrillary GN after treatment with ACTHar gel alone OR in combination with oral tacrolimus (time frame: 12 months)
Experience with H. P. acthar gel treatment of patients with nephrotic syndrome/proteinuria due to various etiologies and its effect on podocyte function (acthar)	40	4 treatment periods of 3 months each with 20 units biweekly, 40 units biweekly, and 80 units biweekly, with a tapering period to no drug for the fourth 3-month period. Then one-year follow-up.	Ongoing	The level of proteinuria
Adrenocorticotropic hormone in membranous nephropathy	25	Single group assignment. Acthar will be administered subcutaneously (SC) 80 units for the first week and then 80 units twice weekly.	Ongoing	Remission of proteinuria
Dose-finding pilot study of ACTH in patients with idiopathic membranous nephropathy (MN)	20	Randomized parallel assignment. Receive ACTH at the dose of 40 or 80 units SQ for up to 12 weeks	Completed	(i) Change in proteinuria, LDL cholesterol, HDL, cholesterol, and triglycerides (ii) Change in side effects/toxicity
Treatment with synthetic ACTH in high-risk patients with membranous nephropathy (ACTHiMeN)	20	Single-group assignment intramuscular injections with Tetracosactide hexaacetate (Synacthen Depot) 1 mg/ml. Treatment for 9 months with an increasing dosage from once per 2 weeks to twice a week.	Completed	Attainability of ACTH therapy with intramuscular injections twice a week for a period of 9 months, measured as the percentage of injections that has been received in line with the treatment schedule
Open-label trial of Acthar gel in subjects with moderate to severe active systemic lupus erythematosus (ACTH)	10	Single group assignment. Patients will administer single dose (80 units) of Acthar subcutaneously every day for 10 days (with a possible 5-day dosing rescue).	Completed	SLEDAI-2K score
A dose escalation study of long-acting ACTH gel in membranous nephropathy	10	Randomized parallel assignment: one arm receives 40 units and the second arm 80 units of the ACTH gel subcutaneously, both given in a dose-escalating frequency beginning at once every two weeks escalating to a maximum of twice per week over a total of three months exposed.	Completed	Change in proteinuria from baseline to value at 3 months
Adrenocorticotropic hormone (ACTH) treatment of nephrotic range proteinuria in diabetic nephropathy (NRDN) (ACTH-NRDN)	14	Randomized single group assignment. Patients with nephrotic range proteinuria randomized to receive 16 or 32 units of ACTHar gel SQ every day.	Completed	8 out of 14 patients achieved a complete (*n* = 1) or partial (*n* = 7) remission
Prospective study evaluating the effect of repository corticotropin in the treatment of various nephrotic syndromes (ACTH)	18	Nonrandomized singe group assignment, acthar 80 IU·SQ once a week and titrated up to twice a week.	Completed	Acthar has the same antiproteinuric effects in a wide range of glomerulonephritis as seen with synthetic ACTH (synacthen) in Europe

**Table 3:** A PICO table illustrating the systematic review criterion.

Criteria	Inclusion criteria	Exclusion criteria
Population	≥1 patient	
Intervention	ACTH (natural or synthetic)	
Comparison	Pre-/postintervention	
Outcomes	Change in proteinuria from baseline to postintervention	No follow-up reported
	Lab values of interest: serum creatinine, albumin, eGFR	No data on remission
Study types	Prospective/retrospective	Systematic reviews
	Case studies	Literature reviews

**Table 4:** Quality criteria checklist for studies.

Study	Inclusion and exclusion criteria specified	Specified sample explained	Study type	Randomized control	Demographic characteristics specified	Serum creatine specified	Proteinuria mentioned	eGFR specified	Follow-up reported	Adverse effects detailed
Madan et al. 2016	Y	Y	R	Y	Y	Y	Y	N	Y	Y
Filippone et al. 2016	Y	Y	R	N	Y	Y	Y	N	Y	Y
Bomback et al. 2012	Y	Y	P	N	Y	Y	Y	N	Y	Y
Bomback et al. 2011	N	N	R	N	Y	Y	Y	Y	Y	Y
Lorusso et al. 2015	Y	Y	P	N	Y	Y	Y	Y	Y	Y
Tumlin et al. 2017	Y	Y	P	N	Y	Y	Y	Y	Y	Y
Alhamad et al. 2019	Y	Y	R	N	Y	Y	Y	Y	Y	Y
Hogan et al. 2013	Y	Y	P, R	N	Y	Y	Y	Y	Y	Y
Berg 2013	Y	Y	R	N	Y	Y	Y	N	Y	N
Hladunewich et al. 2014	Y	Y	P	Y	Y	Y	Y	Y	Y	Y
Hofstra et al. 2010	Y	Y	P	Y	Y	Y	Y	Y	Y	Y
Rauen et al. 2009	Y	Y	R	N	Y	N	Y	N	Y	Y
Ponticelli et al. 2006	Y	Y	P	Y	Y	Y	Y	N	Y	Y
Berg et al. 1999	Y	Y	P	Y	Y	Y	Y	Y	Y	Y
Picardi et al. 2004	Y	Y	R	N	Y	N	N	N	Y	Y
Berg and Arnadottir 2004	Y	Y	R	N	Y	Y	Y	N	Y	Y
Khastgir et al. 2015	Y	Y	R	N	Y	N	N	N	N	Y
Fiechtner and Montroy 2014	Y	Y	P	N	N	Y	N	Y	Y	Y
Tumlin et al. 2013	Y	Y	P	Y	Y	Y	Y	Y	Y	Y
Zand et al. 2019	Y	Y	P	Y	Y	Y	Y	Y	Y	Y
Chart 2019	Y	Y	P	Y	Y	Y	Y	Y	Y	Y

N, no; P, prospective; R, retrospective; Y, yes.

**Table 5:** Summary of studies on ACTH in membranous nephropathy.

Study	*N*	Study protocol	Mean initial lab values	Study outcome	Complications
Membranous nephropathy (MN)					

Berg et al. [8]	14	Synthetic ACTH: 1.6 mg/wk for 2 months	SCr: 1.5 mg/dL SrA: 2.3 g/dL·eGFR: 43 mL/min/1.73 m^2^ proteinuria: 4.8 g/day	1 complete response; 13 partial responses	No serious adverse side effects
Ponticelli et al. [14]	10	Synthetic ACTH: 2 mg/wk for 12 months vs alternating methylprednisolone/cyclophosphamide every month	Proteinuria: 6 g/day	Proteinuria: 0.03 g/day 8 complete remissions; 6 partial responses	Leukopenia (*n* = 2)
Lorusso et al. [15]	9	Synthetic ACTH: 1 mg/wk for 12 months	SrA: 3.21 g/dL proteinuria: 5.3 g/day	SrA: 3.83 g/dL proteinuria: 1.52 g/day 4 complete remissions	N/A
Van De Logt et al. [16]	11	Synthetic ACTH: 2 mg/wk for 9 months	SCr: 104 *μ*mol/L SrA: 22 +/− 6.9 g/L eGFR: 62 mL/min/1.73 m^2^ proteinuria: 8.7 mg/d	eGFR: 72 mL/min/1.73m^2^ proteinuria: 2 mg/d 4 complete ones 7 partial responses	Mood disorders (*n* = 8), increasing edema (*n* = 11), myalgia (*n* = 7), fever/infection (*n* = 9), hyperpigmentation skin (*n* = 8), hypokalemia (*n* = 4), cushingoid face (*n* = 4)
Picardi et al. [17]	7	Synthetic ACTH: 2 mg/wk for 12 months	N/A	5 complete responses	N/A
Berg and Arnadottir [7]	10	Synthetic ACTH: 1 mg/wk or dose based on body weight (0.5–1 mg once a week or 0.75–1 mg twice a week)	SCr: 1.2 mg/dL proteinuria: 8.38 g/day	Proteinuria: 4.58 g/day 1 complete response 1 partial response	None
Rauen et al. [18]	4	Synthetic ACTH: 0.25–2.25 mg/wk	eGFR: 40.2 mL/min/1.73 m^2^ proteinuria: 11.2 g/day	2 complete responses 2 partial responses	(*n* = 1) weight gain, elevated blood pressure, and hyperglycemia
Hladunewich et al. [19]	20	Acthar gel: 40 or 80 units/wk	Proteinuria: 9068 ± 3384 mg/day SrA: 2.72 ± 0.83 g/dl	Proteinuria: 3866 ± 4243 mg/day SrA: 3.25 ± 0.60 g/dL 2 complete remissions 10 partial remissions	Cushingoid appearance (*n* = 3)
Chart study [20]	60	Double blind randomized trial. Patients either received 40 units 5x a week or 80 units 2x a week	N/A	1 partial remission in 40 U arm; 5 partial remissions in 80-unit arm and 2 partial ones in combined placebo	Pneumonia (*n* = 2) generalized edema (*n* = 1) drug hypersensitivity (*n* = 1)
Madan et al. [2]	11	Acthar gel: 80 units twice weekly for 6 months	SrA:1.73–3.9 g/dl SCr: 0.9–4.8 mg/dl Proteinuria: 2500–9306 mg/d (median: 5000)	61.1% change in proteinuria levels 4 complete remissions 2 partial remissions	1 early termination due to fatigue and dizziness
Bomback et al. [5]	11	Acthar gel: 160 units/week for 6 months	Proteinuria: 6827 mg/day	Proteinuria: 2852 mg/day 3 complete remissions 6 partial remissions	*n* = 2, hyperglycemia *n* = 1, bone demineralization *n* = 1, weight gain
Bomback et al. [4]	5	Subcutaneous acthar gel 40 units BIW for 2 week then 80 units BIW for 24 weeks	Proteinuria: 5.65 mg/g SCr: 1.7–2.9 mg/dl SrA: 2.1–4.1 g/dl	Proteinuria: 4.42 mg/g SCr: 1.2–1.9 mg/dl 2 partial remissions	*n* = 2, worsened glycemic control *n* = 1, increase in skin pigmentation

ACTH, adrenocorticotropic hormone; eGFR, estimated glomerular filtration rate; SCr, serum creatinine; SrA, serum albumin.

**Table 6:** Summary of studies on ACTH in focal segmental glomerulosclerosis.

Study	*N*	Study protocol	Mean initial lab values	Study outcome	Complications
Focal segmental glomerulosclerosis (FSGS)					

Madan et al. [2]	15	Acthar gel: 40–80 units every 24 to 72 hours for minimum of 24 weeks	SrA: 1.7–3.9 g/dL (3.17 ± 0.54) SCr: 0.9–4.8 mg/dL, 2.27 ± 1.16 proteinuria: 2500–9306 mg/d (5238 ± 1975 mg/d)	SrA: 2.7–4.0 g/dl (3.56 ± 0.13) SCr: 0.8–6.7 mg/dl (1.77 ± 0.64) proteinuria: 750–3560 mg/d (2216 ± 1000 mg/d) Mean 57.7% reduction in proteinuria 9 partial remissions	Increased swelling *n* = 1; Hypoalbuminemia in 8/15 patients; improvement in 7 by posttreatment; 2 early terminations
Tumlin et al. [21]	13	Acthar gel: 40–80 units, 2 to 3 times per week for 6 months	SCr: 1.8 ± 0.2 mg/dL eGFR: 47 ± 6.8 mL/min/1.73m^2^ UP/Cr ratio: 7.92 ± 1.1 g/g Proteinuria: 6.47 ± 1.2 g/g	eGFR: 52.3 ± 8 mL/min/1.73 m^2^ UPCR ratio: 2.98 ± 0.6 g/g Proteinuria: 1.51 ± 0.7 g/g 1 complete remission 8 partial responses	Hyperglycemia *n* = 5 Reduction on Acthar *n* = 1
Hogan et. al. [22]	24	Acthar gel: *n* = 12: 40 u weekly for 2 weeks then 80 units weekly for 2 weeks 80 units biweekly for 12 weeks *n* = 7: 40 units biweekly for 2 weeks 80 units biweekly for 22 weeks *n* = 5: 80 units SC twice weekly	Median proteinuria: 4595 mg/g (IQR, 2200–8020) SCr: 2.0 mg/dL (IQR, 1.1–2.7) eGFR: 36 mL/min per 1.73 m^2^ (IQR, 28–78)	Median proteinuria: 2243 mg/g (IQR, 1570–5620) SCr: 1.4 mg/dL (IQR, 1.1–2.0) eGFR: 45 mL/min per 1.73 m^2^ (IQR, 28–74) 5 partial remissions 2 complete remissions	21 patients had adverse effects: swelling (*n* = 5), mood alteration (*n* = 4), elevated blood pressure (*n* = 3), dyspepsia (*n* = 2), hyperglycemia (*n* = 1), upper respiratory infection (*n* = 4), muscle cramps (*n* = 4), polyurea (*n* = 1), rash (*n* = 2).
Alhamad et al. [23]	20	Acthar gel: 80 units twice a week for 6 months	SCr: 3.3 ± 2.7 mg/dl SrA: 3.6 g/g eGFR: 30.7 ± 19.3 mL/min/1.73 m^2^ proteinuria: 8.8 ± 7.6 g/g	SCr: 2.8 ± 1.67 mg/dl SrA: 3.7 g/g eGFR: 34.4 ± 20 mL/min/1.73 m^2^ proteinuria: 3.3 ± 2.3 g/g 4 complete remissions 6 partial remissions	8 reports of graft failure
Bomback et al. [5]	1	Acthar gel: 80 units twice a week for 6 months	Proteinuria: 10275 mg/day	Proteinuria: 2970 mg/day 1 partial remission	None
Bomback et al. [4]	3	Acthar gel: 40 units twice a week for 2 week and 80 units twice a week for 24 weeks	SCr: 1.0 mg/dL SrA:3.37 g/dL UPCR: 1.85 g/g Proteinuria: 1.85 g/g	SCr: 1.13 mg/dL SrA: 3.33 g/dL UPCR: 2.15 g/g Proteinuria: 2.14 g/g 1 partial remission	Temporary increase in skin pigmentation *n* = 1
Fillippone et al. [3]	10	Acthar gel: 40 units weekly (*n* = 2) or 80 units twice weekly (*n* = 8)	SCr: 2.22 mg/dL Proteinuria: 5.8–8.43 g/d	SCr: 2.12 mg/dL Proteinuria:6 observed reduction in proteinuria 2 partial remissions 2 complete remissions	1 patient withdrew due to side effects Weight gain *n* = 4 Myalgia *n* = 2 Worsening diabetes *n* = 2 Hypertension *n* = 2 Edema *n* = 1
Berg et al. [24]	10	Synthetic ACTH: 1 mg once a week for 6 months	Proteinuria: 3400 mg/d	Proteinuria: 1700 mg/d 1 partial remission	N/A
Lorusso et al. [15]	2	Synthetic ACTH: 1 mg once a week for 12 months	SrA: 2.5 g/dL Proteinuria: 18 g/d	SrA: 3 g/dL Proteinuria: 0.93 g/d	N/A

eGFR, estimated glomerular filtration rate; SCr, serum creatinine; SrA, serum albumin; UPCR, urine-protein-creatinine ratio.

**Table 7:** Summary of studies on ACTH in minimal change disease.

Study	*N*	Study protocol	Mean initial lab values	Study outcome	Complications
Minimal change disease (MCD)					

Madan et al. [2]	2	Acthar gel: 80 units twice weekly for 6 months.	SrA: 2.1–3.7 g/dL SCr: 0.9–1.0 mg/dL Proteinuria: 2000–1500 mg/d	SrA:2.3–4.7 g/dL SCr: 0.7–1.2 mg/dL Proteinuria: 89–241 mg/d 98.1% reduction in proteinuria 2 complete remissions	None
Filippone, et al. [3]	3	Acthar gel: 40 units four times a week (*n* = 2) or 80 units twice weekly (*n* = 8)	SCr: 0.7–1.3 mg/dL Proteinuria: 3.2–12.4 g/g	SCr: 0.51–1.39 mg/dL Proteinuria: 0.270–0.918 g/g 2 partial remissions 1 complete remission	None
Bomback et al. [4]	2	Acthar gel: 40 units twice weekly for 2 week then 80 units twice weekly for 24 weeks	SCr: 0.6–0.7 mg/dL SrA: 2.5–3.4 g/dL UPCR: 3.16–4.76 g/g	SCr: 0.5–0.6 mg/dL SrA: 2.4–3.7 g/dL UPCR: 0.78–11.35 g/g 1 partial remission	None
Bomback et al [5]	1	Acthar gel: 80 units twice weekly for 4 months	Proteinuria: 18,553 mg/day	Proteinuria: 18,557 mg/day	None
Khastgir et al. [6]	2	Acthar gel: 80 units twice weekly for 6 months	N/A	2 complete remissions	N/A
Berg and Arnadottir [7]	2	Synthetic ACTH: 1 mg/wk or dose based on body weight (0.5/1 mg once a week or 0.75/1 mg twice a week)	Proteinuria: 6747 mg/day	Proteinuria: 277.5 mg/day 2 complete remissions	None
Lorusso et al [15]	2	Synthetic ACTH: 1 mg once a week for 12 months	SrA: 2.4–3.4 g/dL Proteinuria: 3.0–3.9 g/d	SrA: 2.5–4.2 g/dL Proteinuria: 0.5–6.3 g/d *n* = 1 complete remission	None

ACTH, adrenocorticotropic hormone; eGFR, estimated glomerular filtration rate; SCr, serum creatinine; SrA, serum albumin; UPCR, urine protein creatinine ratio.

**Table 8:** Summary of studies on ACTH in lupus nephritis.

Study	*N*	Study protocol	Mean initial lab values	Study outcome	Complications
Lupus nephritis (LN)					

Khastgir et al. [6]	2	Acthar gel: 80 units twice weekly for 6 months	N/A	2 partial remissions	None
Madan et al. [2]	2	Acthar gel: 80 units twice weekly for 6 months	SCr: 1.0 mg/dl SrA: 1.7–1.8 g/dl Proteinuria: 8000–19890 mg/d	SCr: 0.8–1/1 mg/dl SrA: 2.4–3.3 g/dl Proteinuria: 1089–2454 mg/d 87.3% reduction in proteinuria 2 partial remissions	None
Fiechtner and Montroy [26]	10	Acthar gel: 80 units per day for 7–15 days	N/A	Improvement in joint and active skin problems	Edema (*n* = 1)
Bomback et al. [5]	1	Acthar gel: 40 units thrice weekly for 5 months	Proteinuria: 1340 mg/day	Proteinuria: 2290 mg/day No response	Weight gain (*n* = 1)

SCr, serum creatinine; SrA, serum albumin.

**Table 9:** Summary of studies on ACTH in IgA nephropathy.

Study	*N*	Study protocol	Mean initial lab values	Study outcome	Complications
IgA nephropathy (IgAN)					

Bomback et al. [5]	1	Acthar gel: 40 units twice per week for 8 months	Proteinuria: 4952 mg/day	Proteinuria: 42 mg/day 1 complete response	None
Bomback et al. [4]	5	Acthar gel: 40 units twice per week for 2 weeks then 80 units twice per week for 6 months	SCr: 0.8–2.7 mg/dl SrA: 3.7–4.3 g/dl UPCR: 0.61–1.95 g/g	SCr: 0.8–2.1 mg/dl SrA: 4.1–4.5 g/dl UPCR: 0.21–1.22 g/g 1 complete response 1 partial response	*n* = 1 weight gain, cushingoid facies, increased blood pressure; Withdrawn due to worsening kidney function
Madan et al. [2]	5	Acthar gel: 80 units twice weekly for 6 months	SCr: 1.0–2.8 mg/dl SrA: 3.0–2.0 g/dl Proteinuria 2500–9306 mg/d (4268 ± 2931 mg/d)	SCr: 1.0–1.5 mg/dl SrA: 3.6–4.2 g/dl Proteinuria: 800–2360 mg/d (1416 ± 654 mg/d) 67.3% reduction in proteinuria 2 partial remissions	1 early termination (*n* = 1) weight gain (*n* = 1) hypertension
Khastgir et al. [6]	5	Acthar gel: 80 units twice weekly for 6 months	N/A	2 partial remissions 36.4% proteinuria reduction	1 early termination due to hypertension and weight gain
Zand et al. [24]	19	Acthar gel: 80 units twice weekly for 6 months	SCr: 1.40 ± 0.49 mg/dl SrA: 3.79 ± 0.54 g/dl 24 hr UP: 2635 (1230–5243) mg	SCr: 1.55 ± 0.64 mg/dl SrA: 3.93 ± 0.39 g/dl 24 hr UP: 1274 (344–6228) mg 8 partial remissions	6 infections: 2 viral, 2 sinusitis, 1 pneumonia, 1 otitis media

SCr, serum creatinine; SrA, serum albumin; UP, urine protein; UPCR, urine protein creatinine ratio.

**Table 10:** Summary of studies on ACTH in membranoproliferative glomerulonephritis.

Study	*N*	Study protocol	Mean initial lab values	Study outcome	Complications
Membranoproliferative glomerulonephritis (MPGN)					

Berg and Arnadottir [7]	6	Synthetic ACTH: 1 mg/wk or dose based on body weight (0.5/1 mg once a week or 0.75/1 mg twice a week)	Proteinuria: 5027–26660 mg/d (12,041 ± 7806 mg/d)	Proteinuria: 56–762 mg/day (392 ± 304 mg/d) 6 complete responses	N/A
Lorusso et al. [15]	2	Synthetic ACTH: 1 mg/wk for 12 months	SrA: 2.6–2.7 mg/dL Proteinuria: 10–16 g/day (13 ± 3 g/d)	SrA: 4.4–4.5 mg/dL Proteinuria: 0.8–10.8 g/day (5.8 ± 5 g/d) 1 complete remission	1 early termination
Bomback et al. [5]	4	Acthar gel: 80 units twice per week (*n* = 3) or 40 units three times per week (*n* = 1) for 4–6 months	Proteinuria (80 U): 5500–13073 mg/d(9605 ± 3124 mg/d) Proteinuria (40 U): 12398 mg/day	Proteinuria (80): 3741–4825 mg/d(4148 ± 481 mg/d) 3 no response Proteinuria (40): 4560 mg/d Limited response	None
Madan et al. [2]	1	Patients received 80 U acthar gel twice weekly for 6 months	SCr: 0.7 mg/dl SrA: 1.5 g/dl Proteinuria 10000 mg/d	SCr: 0.8 mg/dl SrA: 3.3 g/dl Proteinuria: 2141 mg/d *n* = 1 Partial remission	None

ACTH, adrenocorticotropic hormone; SCr, serum creatinine; SrA, serum albumin.

## Data Availability

The data used in this study are all published online.
